# Accuracy and reliability of three-dimensional computer-assisted planning for orthognathic surgery

**DOI:** 10.1186/s40902-018-0154-4

**Published:** 2018-06-25

**Authors:** Tae-Geon Kwon

**Affiliations:** Department of Oral & Maxillofacial Surgery, School of Dentistry, Kyungpook National University, Samduck 2 Ga, Jung Gu, Daegu, 700-421 South Korea

Recent wide-spread application of three-dimensional (3D) computerized planning has changed several aspects in clinical protocols for orthognathic surgery. Cone-beam computed tomography (CBCT), 3D image software, and 3D printing equipment have become necessary tools for application in computer-assisted orthognathic surgery [1]. Three-dimensional virtual surgical planning is regarded as an accurate and reliable method that is comparable to conventional two-dimensional (2D) frontal and lateral cephalography [2].

To reduce discrepancy in surgical planning, errors associated with planning and surgical process need to be minimized. The errors are divided into two categories: accuracy and reproducibility [3]. Sometimes these two types of the errors cannot be differentiated. Differentiation between the two types of error may not always be possible. However, a thorough understanding is required of the number of errors that are possibly related to computerized surgery.

Accuracy, also referred to as validity, represents deviation from the actual or original value. Usually, it reflects errors in the treatment system itself. In the 3D computerized planning process, errors in surface rendering (thresholding), data integration (data merge from dentition to CT), and settings for 3D coordinates in the virtual space or splint fabrication (3D printing process) are related with accuracy. It can be improved by systemic process in surgical planning and preparation. Despite changes in soft tissue due to skeletal movements, the differences in various components of soft tissues cannot be completely computed for individual patients [4]. Therefore, every possible error related to the computer algorithm of soft tissue simulation can also be regarded as systemic error.

Reproducibility or reliability refers to the closeness of repeated or successive measurements, which depend on the examiner’s skill or surgeon’s condition, and are classified as random errors. In general, studies using cephalometric measurements use the Dahlberg’s formula to determine the presence of this type of error [3]. During surgery under 3D computerized planning, errors in landmark identification, planning process itself, and segment positioning during the fixation can undermine the precision of the surgery, since the intraoperative maxillary position greatly depends on the intermediate splint fabricated from the model surgery; hence, this step is important for accurate maxillary positioning in conventional orthognathic surgery. Face-bow transfer, mounting of a dental cast onto the articulator, and the model surgery procedure are potential sources of random errors. Recent application of computer-assisted 3D virtual model surgery and 3D-printed intermediate occlusal splint is a useful method but not completely; nevertheless, it is not free from random error.

Many studies have shown the advantages of virtual 3D surgical planning and application of computer-aided surgical procedure [5]. According to previous reports, the accuracy of 3D computer-assisted orthognathic surgery was “comparable to” but not significantly higher than that of the conventional orthognathic procedure [2, 6]. A recent report suggested that 3D computerized planning cannot completely replace real-time monitoring in the operation theater and “some margin for correction of inaccuracies” is required in computerized planning [6]. Minimizing the systemic errors such as integration of 3D dentition to 3D facial CT and integration of 3D skin to 3D skull CT data is required. To increase the reliability or reproducibility, computer-aided surgical simulation using splint and surgical guide and determination of occlusion in virtual space need to be improved further. Concurrently, any hidden costs or limitations in virtual 3D surgical simulation in orthognathic surgery need to be clearly understood.
